# Current updates on the molecular genetics and magnetic resonance imaging of focal nodular hyperplasia and hepatocellular adenoma

**DOI:** 10.1007/s13244-015-0399-8

**Published:** 2015-03-20

**Authors:** Maneesh Khanna, Subramaniyan Ramanathan, Najla Fasih, Nicola Schieda, Vivek Virmani, Matthew D. F. McInnes

**Affiliations:** 1 Department of Diagnostic Imaging, The Ottawa Hospital, University of Ottawa, Ontario, Canada; 2 Department of Radiology, Hamad General Hospital, Hamad Medical Corporation, Doha, Qatar; 3 Department of Radiology, Al-Wakra Hospital, Hamad Medical Corporation, Doha, Qatar; 4 Al-Wakra Hospital, Hamad Medical Corporation, PO Box 82228, Doha, Qatar

**Keywords:** Focal nodular hyperplasia, Hepatocellular adenoma, MRI, Molecular genetics, Immunohistochemistry

## Abstract

**Abstract:**

Focal nodular hyperplasia (FNH) and hepatocellular adenomas (HCAs) constitute benign hepatic neoplasms in adults. HCAs are monoclonal neoplasms characterised by an increased predilection to haemorrhage and also malignant transformation. On the other hand, FNH is a polyclonal tumour-like lesion that occurs in response to increased perfusion and has an uneventful clinical course. Recent advances in molecular genetics and genotype-phenotype correlation in these hepatocellular neoplasms have enabled a new classification system. FNHs are classified into the typical and atypical types based on histomorphological and imaging features. HCAs have been categorised into four subtypes: (1) HCAs with HNF-1α mutations are diffusely steatotic, do not undergo malignant transformation, and are associated with familial diabetes or adenomatosis. (2) Inflammatory HCAs are hypervascular with marked peliosis and a tendency to bleed. They are associated with obesity, alcohol and hepatic steatosis. (3) HCAs with β-catenin mutations are associated with male hormone administration and glycogen storage disease, frequently undergo malignant transformation and may simulate hepatocellular carcinoma on imaging. (4) The final type is unclassified HCAs. Each of these except the unclassified subtype has a few distinct imaging features, often enabling reasonably accurate diagnosis. Biopsy with immunohistochemical analysis is helpful in difficult cases and has strong implications for patient management.

***Teaching points*:**

*• FNHs are benign polyclonal neoplasms with no risk of haemorrhage or malignancy.*

*• HCAs are benign monoclonal neoplasms classified into four subtypes based on immunohistochemistry.*

*• Inflammatory HCAs show an atoll sign with a risk of bleeding and malignant transformation.*

*• HNF-1α HCAs are steatotic HCAs with minimal complications and the best prognosis.*

*• β-Catenin HCA shows variable MRI features and a high risk of malignancy.*

## Introduction

Benign hepatocellular lesions in adults can be divided into two main categories according to their pathogenesis: regenerative lesions, composed mainly of focal nodular hyperplasia (FNH), and neoplastic lesions, corresponding to hepatocellular adenomas (HCAs) [1]. FNH and HCAs are the second and third most common benign liver tumours after haemangiomas, respectively [1, 2]. Accurate imaging differentiation of these two types of lesions is essential as the treatment strategies differ considerably. HCAs can present with bleeding or undergo malignant degeneration and often require surgery whereas FNH is a do-not-touch lesion. The recent identification of various molecular pathways altered in these tumours has significantly increased our knowledge of benign hepatocellular tumorigenesis. Moreover, knowledge of the genotype-phenotype correlation in HCA has helped in establishing a new radio-pathological classification. New immunohistochemical and cytogenetic markers have been identified for differentiating various subtypes of HCA. Various studies have identified specific imaging features corresponding to typical and atypical FNH and for differentiating various subtypes of HCA. In this review, we focus on the recent progress in the understanding of the molecular mechanisms and characteristic cross-sectional imaging features with special emphasis on the role of MRI in these two hepatocellular tumours. We also discuss their role in deciding on the different management strategies after diagnosis.

## Focal nodular hyperplasia (FNH)

FNH is defined as a nodule composed of benign-appearing hepatocytes occurring in a liver that is otherwise histologically normal or nearly normal. It is the second most common benign liver tumour after haemangioma with a prevalence of 0.9 % and commonly occurs in young females (male:female ratio = 1:8) [3]. The majority of FNHs are solitary (80 %), smaller than 5 cm in diameter and occur near the surface of the liver [4]. In contrast to monoclonal HCAs, which frequently bleed, FNHs are polyclonal tumour-like lesions and do not undergo haemorrhage or malignant transformation [5]. Although an association with oral contraceptive use has been speculated, owing to the increased prevalence of these tumours in young women, studies have shown that FNH is not hormonally dependent [6]. Association with vascular diseases such as hereditary haemorrhagic telangiectasia (Rendu-Osler-Weber disease) and congenital absence of the portal vein can be seen [1].

FNH is often an incidental finding at imaging. Distinction between FNH and other hypervascular liver lesions such as HCA, hepatocellular carcinoma (HCC) and hypervascular metastases is critical as the management differs considerably. FNH is asymptomatic in most patients, and in such cases no treatment is necessary. One third of patients may present with abdominal pain or a palpable mass. Typically, FNH follows a benign natural course and remains stable or may even decrease in size at follow-up examination [5].

## Molecular cytogenetics and pathogenesis

Genetic analysis of FNH failed to identify somatic gene mutations in the β-catenin gene (*CTNNB1*), *TP53*, *APC* or HNF1α. FNH typically shows dysregulation of the angiopoietin genes, which are responsible for the maturation of blood vessels. An increase in the angiopoietin expression results in uncontrolled maturation and remodelling of vessels, resulting in dystrophic vascular architecture typical of FNH [1, 7]. Wanless et al. postulated that portal tract injury due to either portal tract inflammation or arterial ischaemia is the primary event leading to vascular shunting with hepatocyte hyperplasia and cholestasis [8, 9]. Arterial hyperperfusion and the resultant hyperoxemia lead to increased expression of vascular endothelial and somatic growth factors and activation of hepatic stellate cells leading to the formation of the characteristic central scar [8].

The β-catenin pathway is activated, including the downstream target, glutamine synthetase, which explains the polyclonal over-proliferation of hepatocytes. The molecular mechanisms of this activation are uncertain, but do not involve demonstrable mutations in β-catenin or Axin, unlike HCA [10]. Immunohistochemistry shows a perivenous map-like distribution of glutamine synthetase [11].

## Classification

Currently, FNH is divided into two types: classic and non-classic or atypical. [12].

## Classic FNH

Classic FNH is characterised by the presence of abnormal nodular architecture, malformed vessels and cholangiolar proliferation. One or more macroscopic central scars are present in most cases that contain fibrous connective tissue, cholangiolar proliferation with surrounding inflammatory infiltrates and malformed vessels. Arterial blood flows centrifugally from the anomalous central arteries. Approximately 50 % of lesions show some degree of fatty infiltration, as opposed to the surrounding liver, which shows signs of steatosis in less than 20 % of lesions. Both the classic and non-classic types contain variable amounts of Kupffer cells [13].

## Imaging features

Classic FNH has typical imaging findings enabling accurate diagnosis. FNH is found as an incidental iso- to hypoechoic focal lesion on ultrasound although the isoechoic nature often makes the detection of lesions difficult. A central vascular scar may be seen. Further evaluation with dynamic contrast-enhanced CT or MRI is warranted in most cases for definitive diagnosis. It appears as a lobulated iso- to hypodense lesion on unenhanced CT and shows homogeneous, intense arterial enhancement becoming isoattenuating in the portal and delayed phases with no washout. The central scar showing delayed enhancement because of myxomatous stroma can be seen in 20 % of cases [14].

**Fig. 1:**
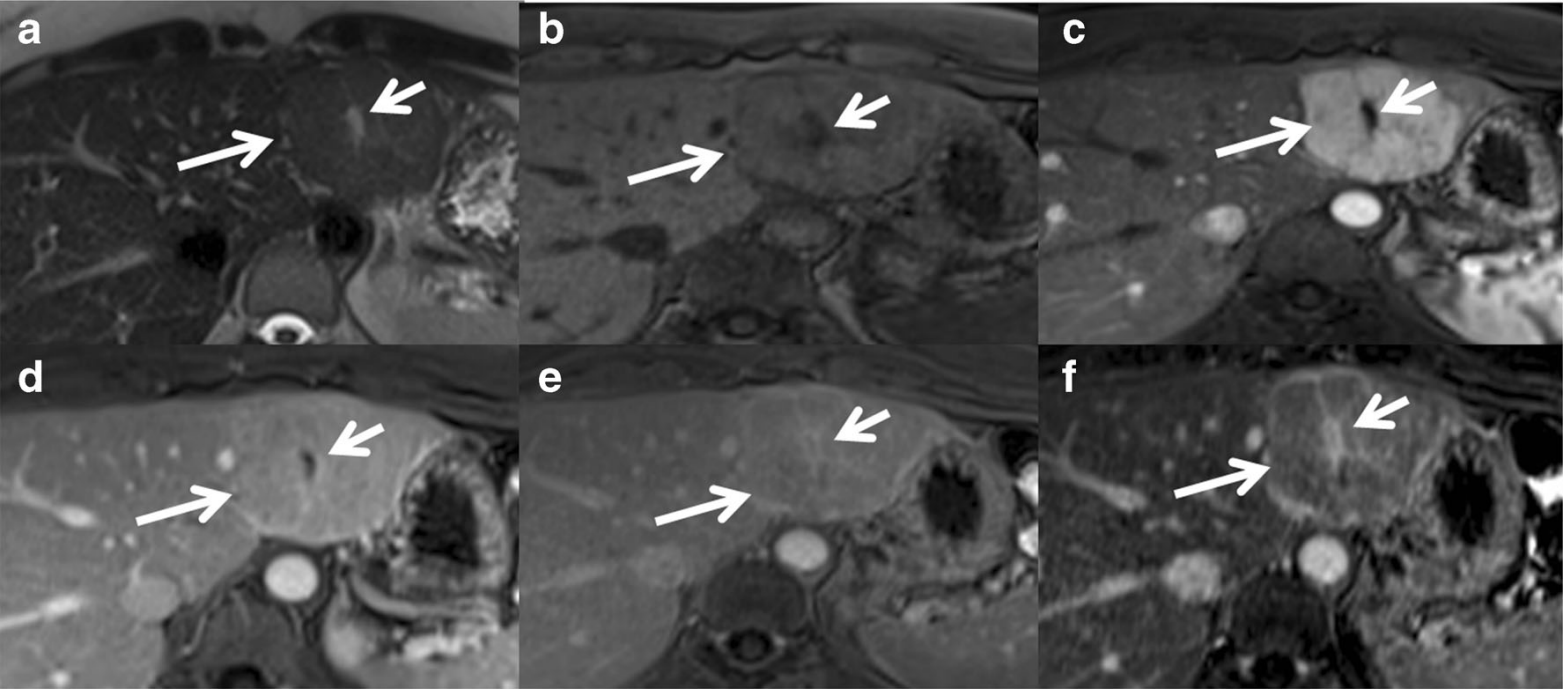
Classic focal nodular hyperplasia (FNH) in a 40-year-old female incidentally detected on ultrasound (not shown here). (**a**) Axial T2W MRI shows a well-circumscribed T2 isointense lesion (*long arrow*) in the left lobe of the liver with a central T2 hyperintense scar (short arrow). (**b**) Axial T1W MRI shows a well-circumscribed T1 isointense lesion (long arrow) in the left lobe of the liver with a central T2 hypointense scar (*short arrow*). (**c**) Axial post-contrast MRI in the arterial phase reveals intense enhancement of the lesion (*long arrow*) with a non-enhancing central scar (*short arrow*). Axial post-contrast MRI in the venous (**d**) and delayed phases (**e** and **f**) shows the isointense nature of the lesion (*long arrow*) with progressive delayed enhancement of the central scar (*short arrow*)

Magnetic resonance imaging (MRI) has higher sensitivity (70 %) and specificity (98 %) for FNH than US and CT. MRI features include T1 iso- or hypointensity (94–100 %), T2 slight hyper- or isointensity (94–100 %) and a hyperintense central scar (84 %). A scar is usually seen in FNH >3 cm [15]. Lesions show intense homogeneous enhancement in the arterial phase, isointensity during the portal phase and delayed enhancement of the central scar [16] (Fig. 1).

**Fig. 2:**
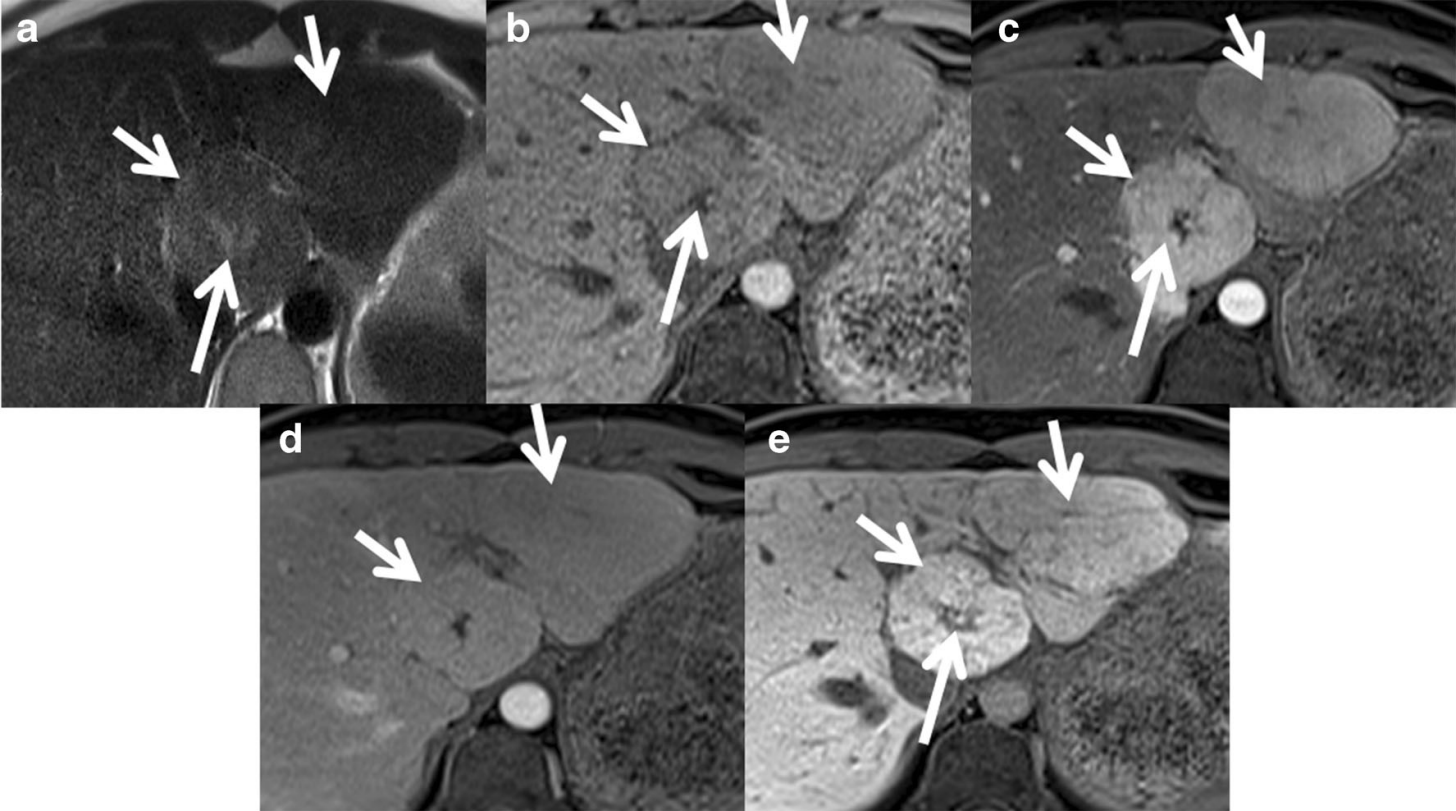
Classic FNH in a 35-year-old female with hepatobiliary-specific contrast (gadoxetate). (**a**) Axial T2W MRI shows two large isointense lesions in the left lobe of the liver (*short arrows*) with a T2 hyperintense central scar (*long arrow*). (**b**) Axial T1W MRI shows two large isointense lesions in the left lobe of the liver (*short arrows*) with a T1 hypointense central scar (*long arrow*). Axial post-contrast MRI with gadoxetate shows intense enhancement of the lesions (short *arrows*) in the arterial phase (**c**) becoming isointense on the portal venous phase (**d**) and persistent enhancement in the 20-min hepatobiliary phase (**e**). The central scar shows no enhancement (*long arrow*)

**Fig. 3:**
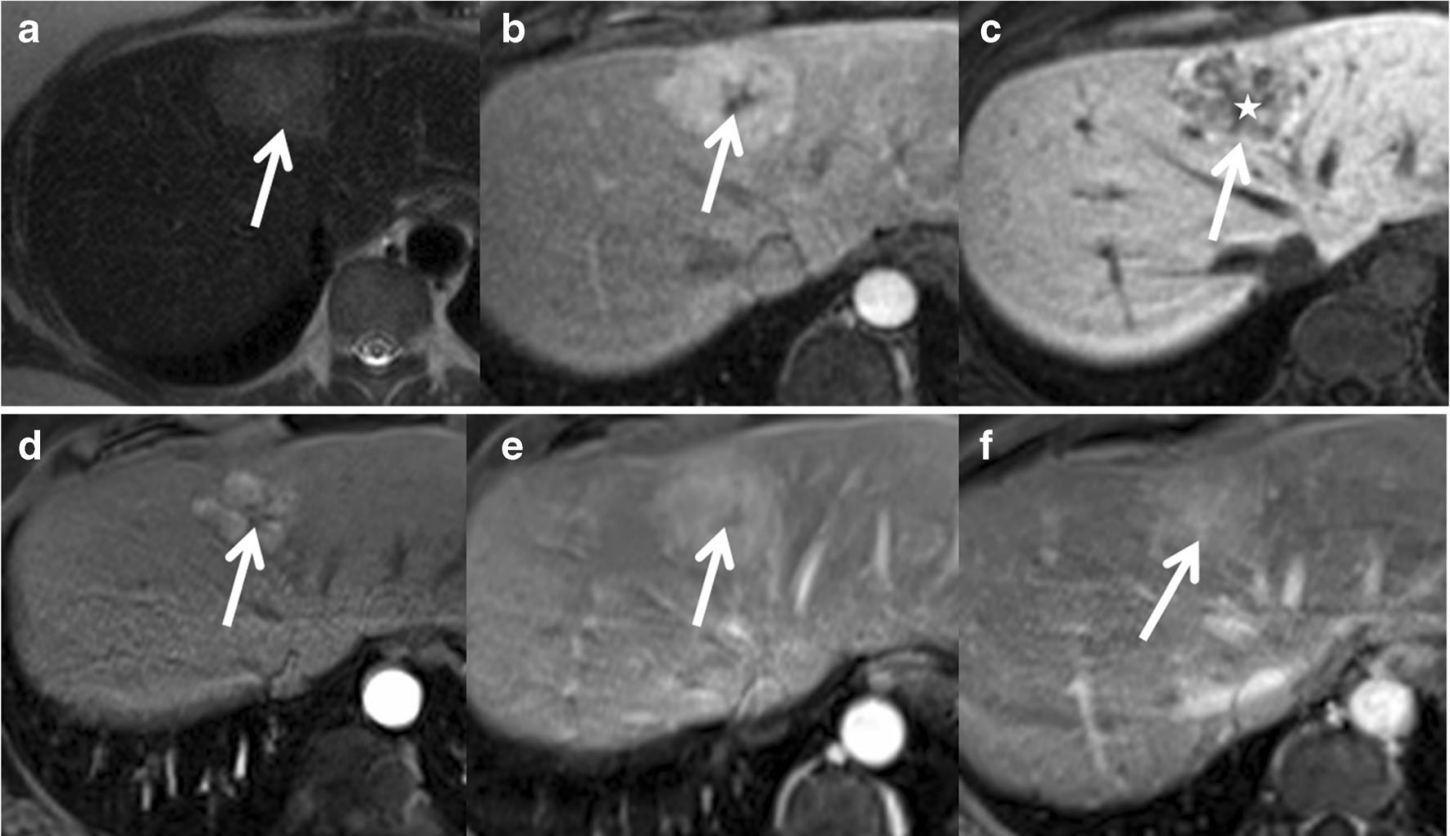
Appearance of FNH with hepatobiliary-specific contrast (gadoxetate) and a routine extracellular agent (gadolinium). (**a**) Axial T2W MRI shows iso- to mildly hyperintense lesions in segment 4 of the left lobe of the liver (*arrow*). (**b**) Axial post-contrast MRI with gadoxetate shows intense enhancement of the lesions in the arterial phase with a non-enhancing central scar (*arrow*). (**c**) Axial post-contrast MRI in the 20-min hepatobiliary phase shows a peripheral ring of hyperintensity (*arrow*) with central non-enhancement (asterisk). Follow-up MRI with gadolinium after 6 months shows an enhancing lesion with a non-enhancing scar (*arrow*) in the arterial phase (**d**) and persistent enhancement in the portal venous (**e**) and delayed phases (**f**) with delayed enhancement of the central scar (*arrow*)

Hepatobiliary-specific contrast agents are being increasingly used in differentiating focal liver lesions. FNH is characterised by an increased density of functioning hepatocytes and hence shows persistent enhancement (iso- or hyperintense) on delayed phase (20 min) gadoxetic acid-enhanced images (Fig. 2). Three patterns of enhancement are described on the 20-min hepatobiliary phase: a homogeneously hyperintense, inhomogeneously hyperintense and peripheral hyperintense rim (hypointense with a ring pattern) (Fig. 3). Approximately 10–12 % of the lesions may not show hyperintensity on the 20-min delayed phase, warranting further evaluation with biopsy or close interval imaging [17]. The presence of abnormal bile ductules that fail to communicate with the normal biliary system possibly results in defective or delayed excretion with persistent contrast agent retention [18, 19]. In contrast, HCA and HCC typically do not show contrast agent retention. A central scar appears hypointense on 10- and 20-min delayed phases because of the predominant fibrous tissue with no well-formed bile ductules [20, 21].

## Non-classic FNH

Non-classic FNH lesions always show cholangiolar proliferation like classic FNH but lack either nodular abnormal architecture or malformed vessels. Lack of a central scar, incomplete multinodular organisation and prominent areas of congestion are some of the histopathological features of non-classic or atypical FNH [22, 12].

## Imaging features

Typical MRI features of FNH lesions, which include homogeneous iso- to hypointensity on TI-weighted images, homogeneous slight hyper- to isointensity on T2-weighted images and a central hyperintense scar on T2-weighted images, are seen in only 9–50 % of these lesions [23, 24]. Various atypical features described at MRI include [16, 25, 26]:

1. Heterogeneous signal intensity on both T1- and T2 weighted sequences due to sinusoidal dilatation, fatty infiltration and/or small haemorrhagic foci.
2. T1 hyperintensity due to fat, copper accumulation, high protein concentrations, blood degradation products or sinusoidal dilatation.
3. Absense of a central scar, which is typically detectable for lesions greater than 3 cm. The scar can be extremely small or undetectable on CT (16–40 %) and MRI (22 %) [4, 14, 27]. The central scar can appear hypointense on T2 with no delayed enhancement mimicking the collagenous scar seen in HCA, fibro-lamellar carcinoma, HCC or intrahepatic cholangiocarcinoma likely from obliterative vascular hyperplasia of the central scar [4, 15].
4. A pseudo-capsule due to compressive effects on the adjacent hepatic parenchyma as well as the presence of dilated vessels and sinusoids around the lesion.
5. Interval growth is considered an atypical and worrisome finding in FNH.
6. FNH can rarely show intralesional steatosis, often due to extension from the underlying hepatic steatosis (Fig. 4). Other possible explanations for intralesional steatosis in FNH are hepatic injury associated with steatosis from alcoholic toxicity, obesity, diabetes or malnutrition [28, 29].

**Fig. 4:**
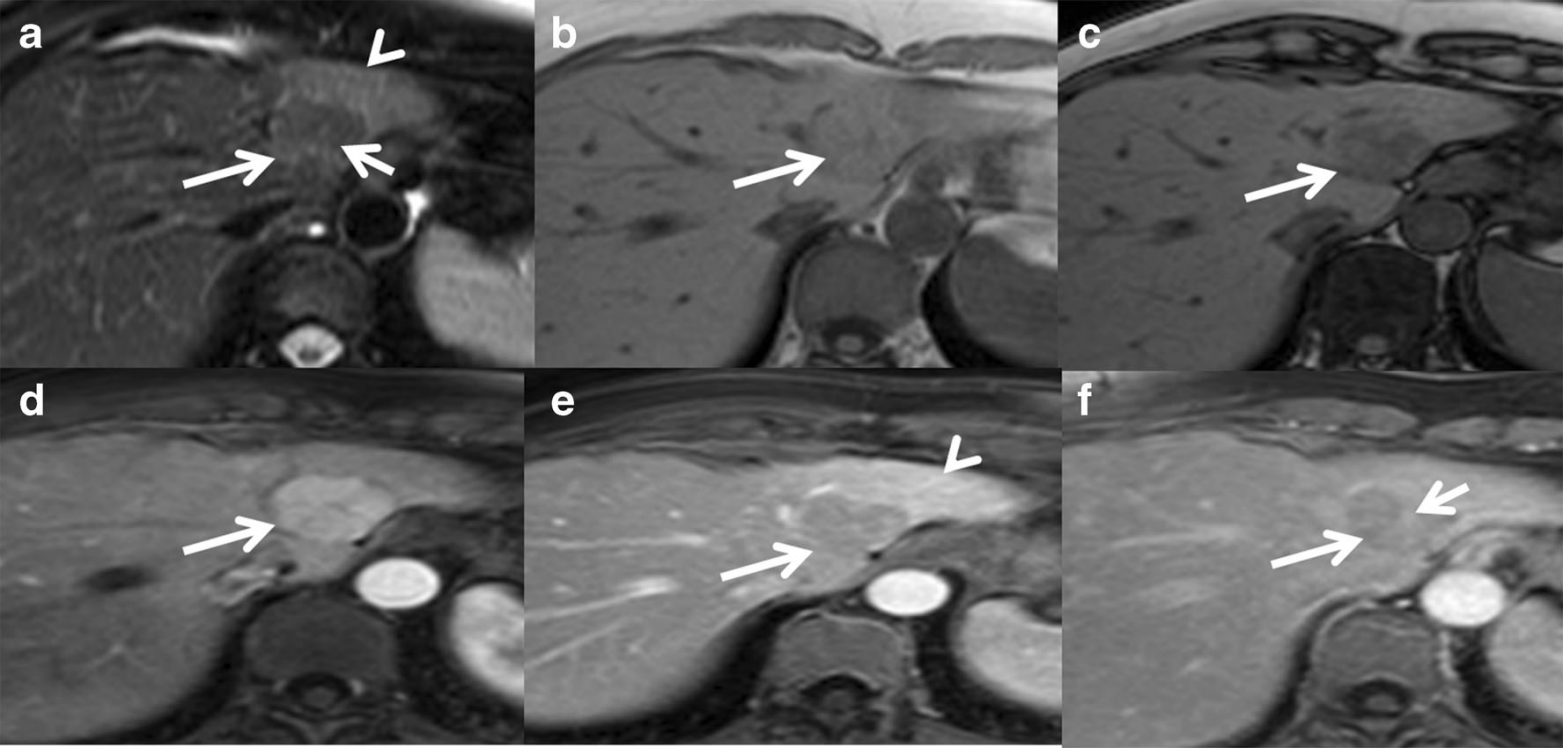
Biopsy-proven FNH with intralesional steatosis in a 46-year-old female. (**a**) Axial T2W MRI shows an isointense lesion (*long arrow*) in the left lobe of the liver with a faint hyperintense central scar (*short arrow*). Axial T1 in-phase (**b**) and out-of-phase (**c**) MRIs show a drop in signal in the out-of-phase images representing intralesional lipid (arrow). Axial post-contrast MRI shows an enhancing lesion (*arrow*) in the arterial phase (**d**) becoming iso- to mildly hypointense in the portal venous (**e**) and delayed phases (**f**) with delayed enhancement of the central scar (*arrow*). Arrowheads denote transient oedema/enhancement
7. Telangiectatic FNH is an uncommon entity but the most common among non-classic FNHs. These lesions often show heterogeneity on pre- and post-contrast CT and MRI, and they are strongly hyperintense on T1- and T2-weighted images, often with an absent central scar. Intense arterial enhancement with persistent enhancement during the hepatic venous and equilibrium phases is seen. Recently, a molecular study identified several genetic similarities between telangiectatic FNH and HCA indicating most of these are actually inflammatory hepatic adenomas and have been misclassified as 'telangiectatic FNH' [30].
8. Development of FNH in childhood and presence of symptoms can also be considered as atypical features.
9. Approximately 20 % of patients have multiple FNHs, which are often of a non-classic subtype with atypical imaging features. Multiple FNH syndrome is defined as consisting of two or more FNHs in combination with hepatic liver haemangioma or vascular malformations or intracranial tumours [31, 32] (Fig. 5). Focal disturbance of the hepatic blood supply has been advocated as the most likely causative factor for the concomitant development of these benign hepatic lesions.

**Fig. 5:**
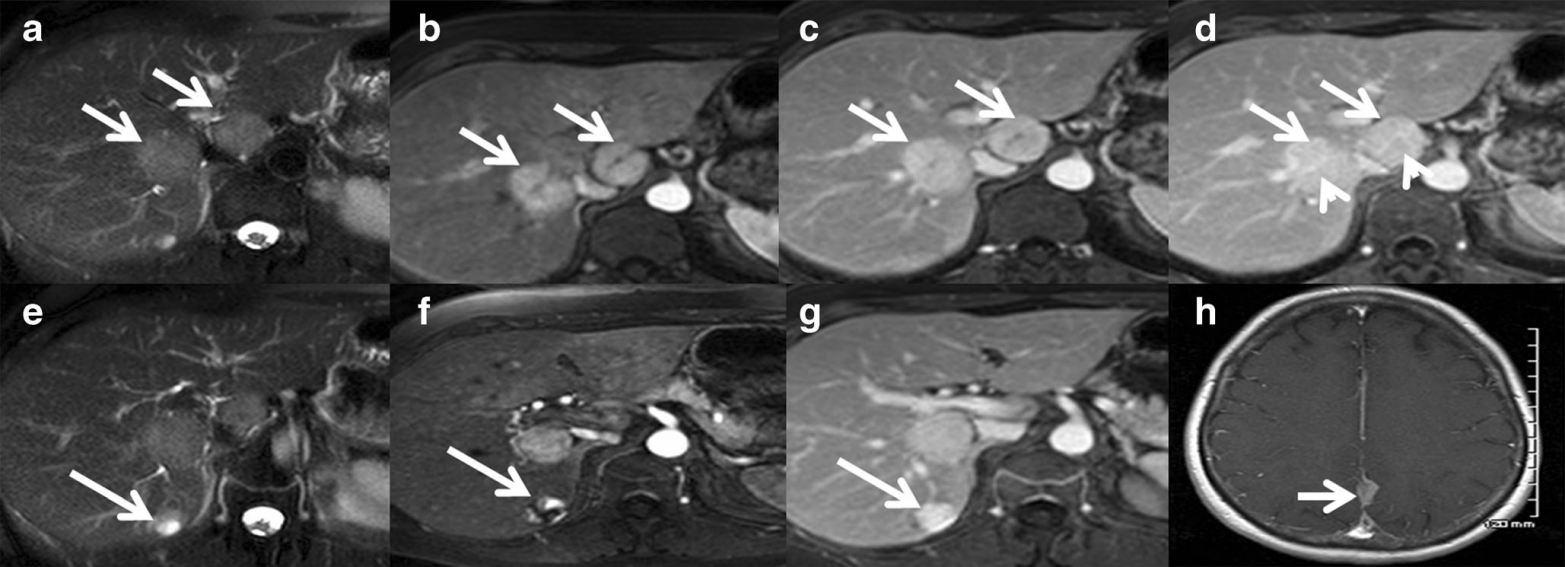
Multiple FNH syndrome in a 50-year-old female. (**a**) Axial T2W MRI shows two iso- to mildly hyperintense focal lesions (*arrows*) in segments 8 and 4 of the liver. Axial post-contrast MRI shows intense enhancement of the lesions (*arrow*) in the arterial phase (**b**) and persistent enhancement (*arrow*) in the portal venous (**c**) and delayed phases (**d**) with delayed enhancement of the central scar (arrowheads). (**e**) Axial T2W MRI in the same patient shows a small T2 hyperintense lesion in segment 7 (*arrow*). Axial post-contrast MRI shows peripheral nodular enhancement (*arrow*) in the arterial phase (**f**) with progressive centripetal filling (*arrow*) in delayed phase (**g**) in keeping with haemangioma. (**h**) Axial post-contrast MRI brain shows a small enhancing extra-axial lesion in the falx cerebri representing meningioma

## Management

FNH with typical imaging features is managed conservatively and does not necessitate surgical intervention. Surgical excision is considered for symptomatic lesions likely from compression of adjacent structures or hepatic capsular stretching. FNH with atypical imaging features needs further evaluation in the form of additional imaging, percutaneous guided biopsy and follow-up. Suspicious lesions could be followed up or surgically excised [33].

## Hepatocellular adenoma (HCA)

HCAs are rare benign monoclonal hepatic tumours that commonly occur in females who have been receiving oral contraceptives. The duration of oral contraceptive use and oestrogen content determine the risk of developing HCA. It rarely occurs in children and men (male:female = 1:9) [34]. Other risk factors for HCAs include use of anabolic steroids, glycogen storage disease (types Ia, III and VI), haemochromatosis, androgen therapy, and use of barbiturates and clomiphene [35]. Patients may present with right upper quadrant pain or a palpable mass. Up to 50 % of patients are asymptomatic. HCAs can be complicated by life-threatening bleeding or undergo malignant transformation necessitating surgical management [36].

## Classification

Initially three MRI patterns were identified corresponding to three pathologic forms, namely the steatotic, peliotic and mixed types [37]. Later, the Bordeaux group divided HCAs into four different subgroups based on molecular genetics, histopathology and clinical features. Various studies have described characteristic imaging features enabling precise diagnosis in select subtypes. Accordingly, HCAs are currently categorised into four distinct genetic and pathologic subtypes: (1) inflammatory HCAs, (2) hepatocyte nuclear factor 1 alpha (HNF-1α)-mutated HCAs, (3) β-catenin-mutated HCAs and (4) unclassified [38, 39].

## Inflammatory HCAs

Inflammatory HCAs are the most common subtype and account for about 40–50 % of all HCAs. Inflammatory HCAs include liver tumours previously referred to as “telangiectatic FNH” or “telangiectatic adenomas”. They occur most frequently in young females with a history of oral contraceptive usage and in obese patients. Patients may present with signs of chronic anaemia and/or systemic inflammatory syndrome characterised by fever, leukocytosis and elevated serum C-reactive protein [40].

Sustained activation of the Janus kinase (JAK)-signal transducer and activator of transcription (STAT) pathway (JAK-STAT pathway), with resultant hepatocellular proliferation, is the proposed pathogenesis in the development of inflammatory HCAs. This can happen via two pathways: (1) somatic gain-of-function mutations involving the IL6ST gene, which encodes the oncogene gp130 in 60 % of cases, and (2) STAT3 activation without mutations in gp130 in 40 % of cases [38]. Often there is activation of acute-phase inflammation proteins, such as serum amyloid A and C-reactive protein [11].

Inflammatory HCAs appear heterogeneous with areas of congestion and frank haemorrhage on gross pathology. Intense polymorph nuclear infiltrates, marked sinusoidal dilatation and thick-walled arteries are seen on histopathology. Immunohistochemistry shows homogeneous glutamine synthetase and β-catenin staining [11].

**Fig. 6:**
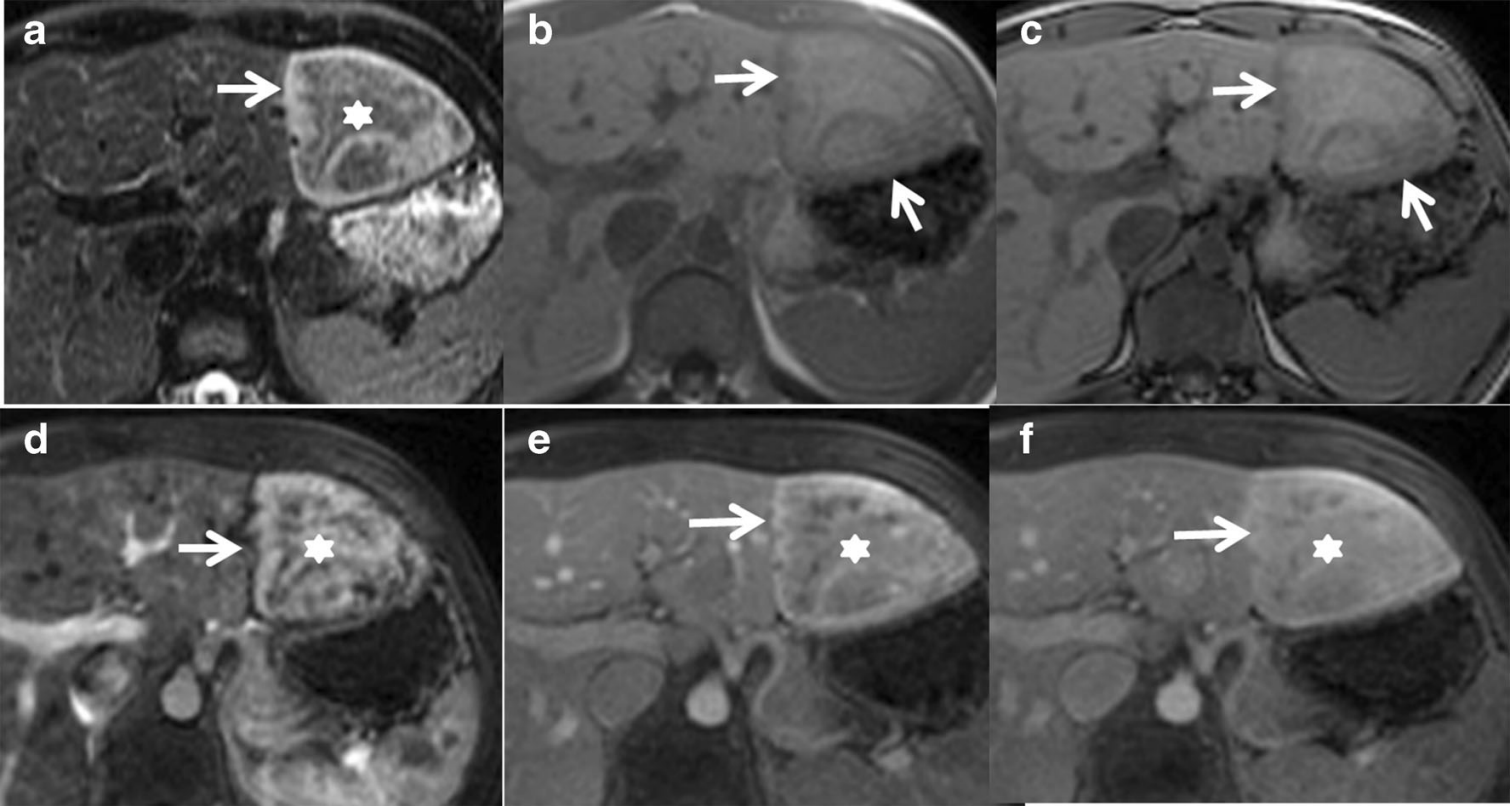
A 30-year-old female with incidental detection of a hypoechoic nodule on ultrasound. (**a**) Axial fat-saturated T2W MRI shows an iso- to hyperintense lesion (asterisk) in the left lobe of the liver with a peripheral T2 hyperintense rim (*arrow*) representing the atoll sign. Axial T1 in-phase (**b**) and out-of-phase (**c**) MRI shows no drop in signal (*arrows*). (**d**) Axial post-contrast MRI in the arterial phase shows moderate heterogeneous central enhancement (asterisk) and peripheral rim enhancement (*arrow*). Axial post-contrast MRI in the venous phase (**e**) and delayed phases (**f**) shows persistent enhancement (asterisk) with delayed enhancement of the peripheral rim (*arrow*). Surgical resection confirmed the diagnosis of inflammatory hepatocellular adenoma

**Fig. 7:**
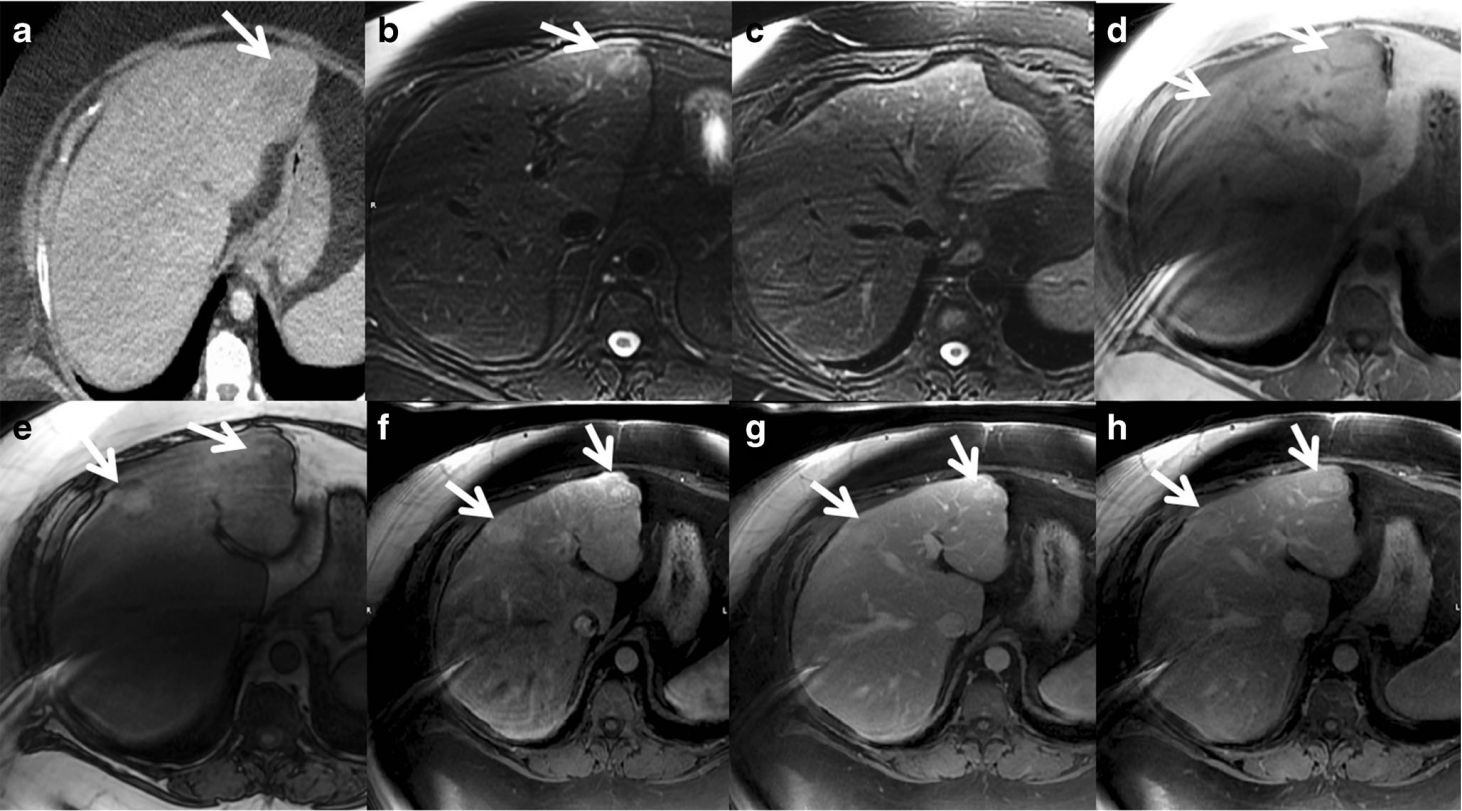
A 38-year-old female with multiple liver lesions. This morbidly obese patient presented with acute abdominal pain and a solitary liver lesion in the lateral segment (*white arrow*) detected on axial contrast-enhanced CT (**a**). Peri-lesional complex fluid was noted (not shown) and the patient was taken to the operative theatre where a haemorrhagic liver lesion was resected. Four other liver lesions were noted at the time of surgery. The clinical and operative presumptive diagnosis was multiple hepatocellular adenomas, most likely of the inflammatory subtype. The original histopathological analysis described the resected lesion as an atypical focal nodular hyperplasia. Immunohistochemistry was performed several months later. Glutaminesynthetase stains depicted typical perivascular staining consistent with adenoma (not shown). **b** Catenin stains were negative and combined with the history of morbid obesity and metabolic syndrome a diagnosis of inflammatory adenoma was given. Follow-up MR examination was performed 2 years after surgery. Axial fat-suppressed T2W FSE images (**b** and **c**) demonstrate two lesions in the lateral segment (*white arrow*) and in the right anterior lobe (*arrowhead*) with increased T2W signal intensity relative to the liver. There is a faint peripheral rim of increased signal in the lateral segment lesion consistent with an atoll sign. Axial T1W images in (**d**) and opposed (**e**) phase demonstrate moderate diffuse hepatic steatosis and inhomogeneous intralesional steatosis (note the peripheral signal intensity drop) within both lesions (*arrows*). Also note the susceptibility artefact on the in-phase GRE image (**d**) at the lateral margin of the left lobe related to previous resection. Axial fat-suppressed T1W GRE images obtained after injection of extracellular gadolinium during the hepatic arterial (**f**), portal venous (**g**) and 5-min delayed (**h**) phases depict heterogeneous arterial enhancement that persists on delayed phases (*arrows*)

On imaging, inflammatory HCAs manifest as hypervascular liver masses with persistent enhancement on portal venous and delayed phase images. They show diffuse T2 hyperintensity and iso- to mild T1 hyperintensity. Focal areas of microscopic fat may be seen in a small (11 %) subset of patients. Sensitivity, specificity, positive and negative predictive values of marked T2 hyperintense signal with delayed persistent enhancement are 85.2, 87.5, and 88.5 and 84 %, respectively, for the diagnosis of inflammatory HCAs [41]. The atoll sign, present in approximately 50 % of cases, includes a T2 hyperintense rim in the periphery of the lesion (correlating with dilated sinusoids) with central isointensity resembling a coral reef and lagoon respectively (Figs. 6 and 7). Small intralesional T2-hyperintense nodules can be found in the centre of the lesion (small islands) [42].

## HNF-1α-mutated HCAs

HNF-1α-mutated HCAs are the second most common subtype and constitute 30–35 % of all HCAs. These develop exclusively in female patients with a history of oral contraceptive use and the tumours are multiple in about 50 % of patients. Often these are incidentally discovered on CT performed for other reasons and are asymptomatic. An association with *maturity*-onset diabetes of the young (MODY), type 3 and familial hepatic adenomatosis has been reported [36, 43, 44].

Biallelic-inactivating mutation of the *TCF1* gene inactivating hepatocyte nuclear factor 1α (HNF-1 α) is the primary event and could be somatic (90–95 %) or germ line (up to 10 %) in origin. The resultant non-functioning HNF-1α protein promotes lipogenesis and hepatocellular proliferation. Also there is suppression of liver fatty acid-binding protein, resulting in impaired fatty acid transport in hepatocytes, leading to intracellular fat deposition. Oestrogens act as endogenous genotoxic agents and cause hepatocyte proliferation by somatic mutations in the TCF1 gene [2, 36, 38].

Excessive lipid accumulation in the tumour hepatocytes is typical on histopathology. No portal tract elements or cytological abnormalities are seen. Immunohistochemistry shows characteristic absence of liver fatty acid-binding protein [11].

**Fig. 8:**
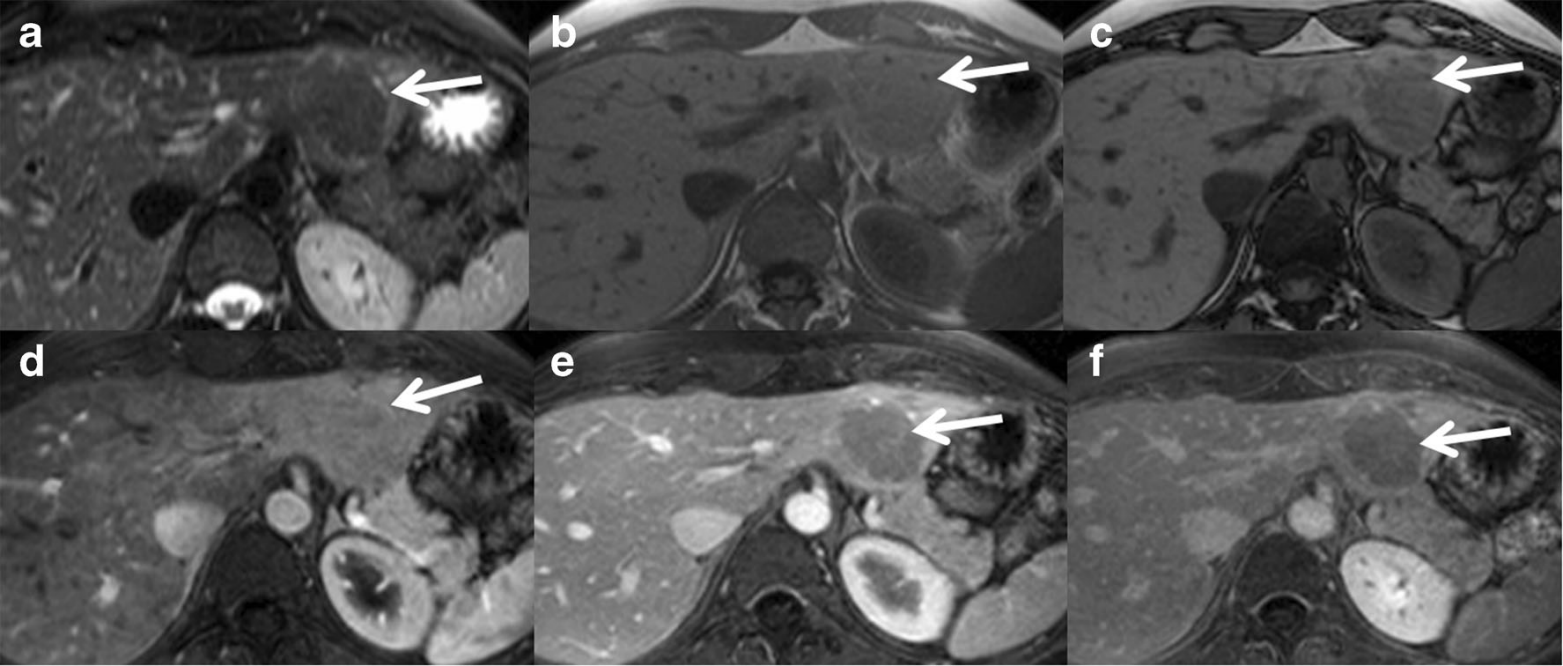
A 26-year-old female with right upper quadrant pain was found to have an echogenic nodule on ultrasound. (**a**) Axial T2W MRI shows an isointense lesion in the left lobe of the liver (*arrow*). Axial T1 in-phase (**b**) and out-of-phase (**c**) MRIs show a drop in signal in the out-of-phase images representing intralesional lipids (*arrow*). Axial post-contrast MRI in the arterial phase (**d**) shows mild diffuse enhancement of the lesion (*arrow*), not persisting in the portal venous (**e**) and delayed phases (**f**). MRI features are consistent with HNF-1α-mutated hepatocellular adenoma and the patient is on regular follow-up

On MRI, these tumours show predominantly hyper- or isointensity on T1 with a diffuse signal drop on chemical shift imaging (CSI) because of intracellular steatosis. T1 hyperintensity could also be due to glycogen and haemorrhage. Iso- to slight hyperintensity is seen on T2. Moderate enhancement in the arterial phase with no persistent enhancement in the portal venous and delayed phases is typical (Fig. 8). Sensitivity, specificity, and positive and negative predictive values of a homogeneous signal drop on CSI for the diagnosis of HNF-1α-mutated HCAs are 86.7, 100, 100 and 94.7 %, respectively [41]. Uncommonly macroscopic fat could be identified on CT in approximately 7 %. MRI is more accurate and shows microscopic fat in 35–77 % of cases [45]. Benign nodular steatosis and fat-containing HCC cannot be completely differentiated from steatotic HCA on imaging alone and may need further evaluation with histopathological and/or immunohistochemical analyses [46]. HNF1α-mutated HCA is the least aggressive subtype and has minimal or no risk of malignant transformation. Tumours <5 cm show minimal risk of bleeding [35].

## β-Catenin-mutated HCAs

β-Catenin-mutated HCAs are the third most common subtype and constitute 10–15 % of all HCAs. These occur more frequently in males and are associated with male hormone administration, glycogen storage disease and familial adenomatosis polyposis [39]. The incidence of HCAs in glycogen storage disease varies from 22–75 and 75 % of these patients older than 30 years harbour HCA. Chronic liver inflammation due to glycogen storage disease leads to the development of HCA and HCC. There is a gain of chromosome 6p and loss of chromosome 6q in these patients, which are also frequently seen in HCC and dysplastic nodules, thereby accounting for the high risk of malignant transformation associated with HCAs in patients with glycogen storage disease [47].

β-Catenin is encoded by the catenin β 1 gene (CTNNB1), located at chromosome 3p21. Mutation results in sustained activation of β-catenin protein, resulting in uncontrolled hepatocyte proliferation. Cytologic abnormalities, such as a high nuclear-cytoplasmic ratio, nuclear atypia and acini formation, are seen on histopathology and mimic well-differentiated hepatocellular carcinoma. Strong and diffuse positivity to glutamine synthase is characteristic of this subtype on immunohistochemical analysis [36].

**Fig. 9:**
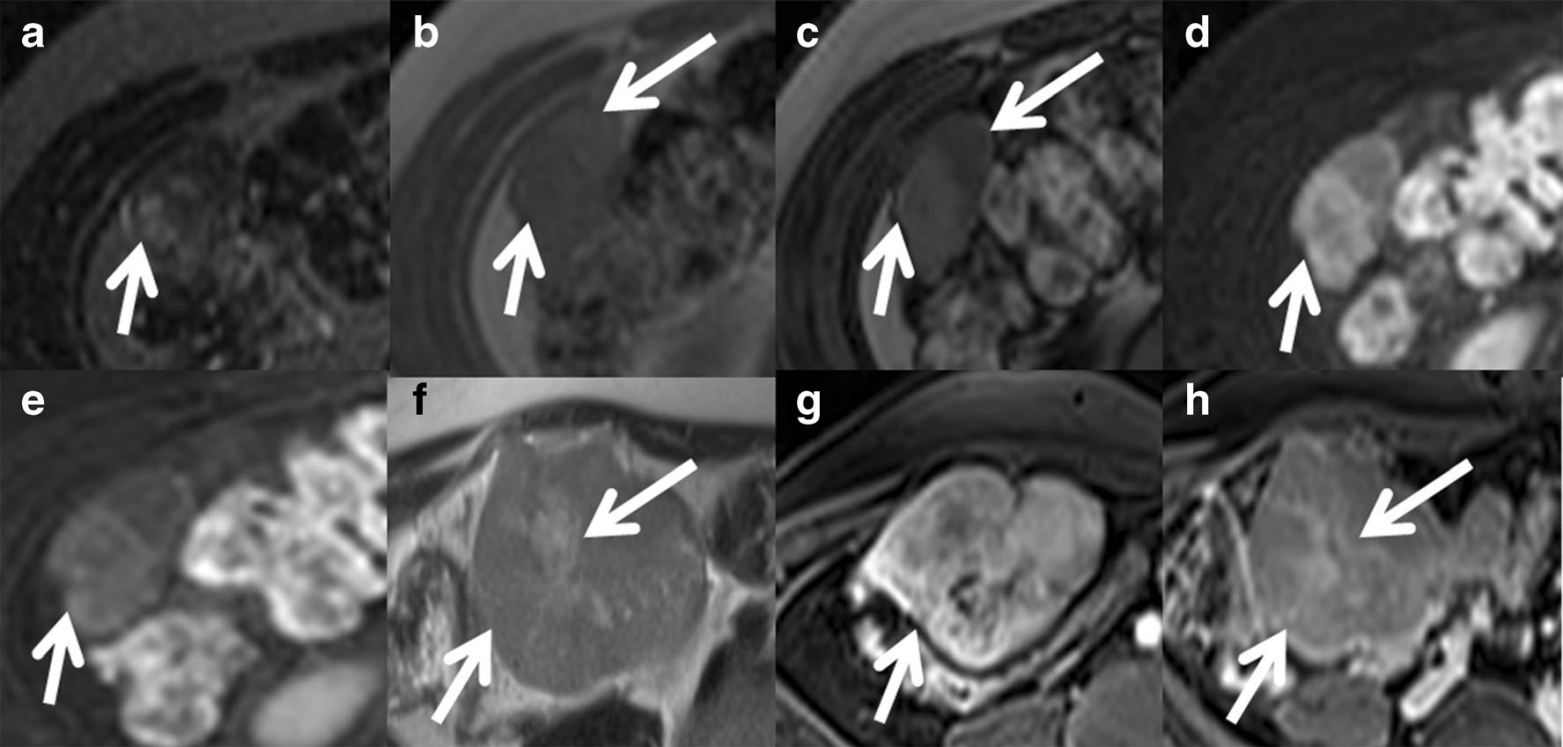
A 42-year-old female with incidental detection of liver nodule. (**a**) Axial T2W MRI shows a small ill-defined lesion in segment 6 with vague T2 hyperintense areas. Axial T1 in-phase (**b**) and out-of-phase (**c**) MRIs show a drop in signal around the lesion indicating perilesional steatosis. Axial post-contrast MRI shows moderate enhancement (*arrows*) in the arterial phase (**d**) becoming iso- to mildly hypointense on delayed phase (**e**). Ultrasound-guided biopsy revealed hepatocellular adenoma (immunohistochemistry and genetic analysis not performed). Follow-up MRI after 2 years showed significant interval growth (*short arrow*) with vague T2 hyperintense areas (*long arrow*) on axial T2W MRI (**f**). Axial post-contrast MRI shows moderate heterogeneous enhancement (*arrow*) in the arterial phase (**g**) becoming iso- to mildly hypointense on delayed phase (**e**) with mild delayed enhancement of a vague central scar (*long arrow*). Surgical resection showed hepatocellular adenoma with multiple foci of well-differentiated hepatocellular carcinoma and genetic analysis revealed β-catenin mutation

On MRI, these tumours show heterogeneous T2 signal, which can be iso-, hypo- or hyperintense relative to the liver and shows no intratumoral steatosis. Intense arterial enhancement is seen, which may or may not persist into the delayed phase. Portal venous washout can be seen mimicking HCC [2]. A recent study correlates the presence of a vaguely demarcated scar and poorly delimited high-signal-intensity areas on T2-weighted images to β-catenin positivity [42] (Fig. 9). Tumours occurring in the setting of glycogen storage disease may show diffuse increased attenuation of liver on CT images.

## Unclassified HCAs

This subtype constitutes approximately 10 % and does not show *HNF1* a, *CTNNB1* or *IL6ST* mutations. The predominant molecular pathogenesis and clinical and radiological features of this subset of tumours are poorly understood and further research is warranted [40].

## Hepatic adenomatosis

**Fig. 10:**
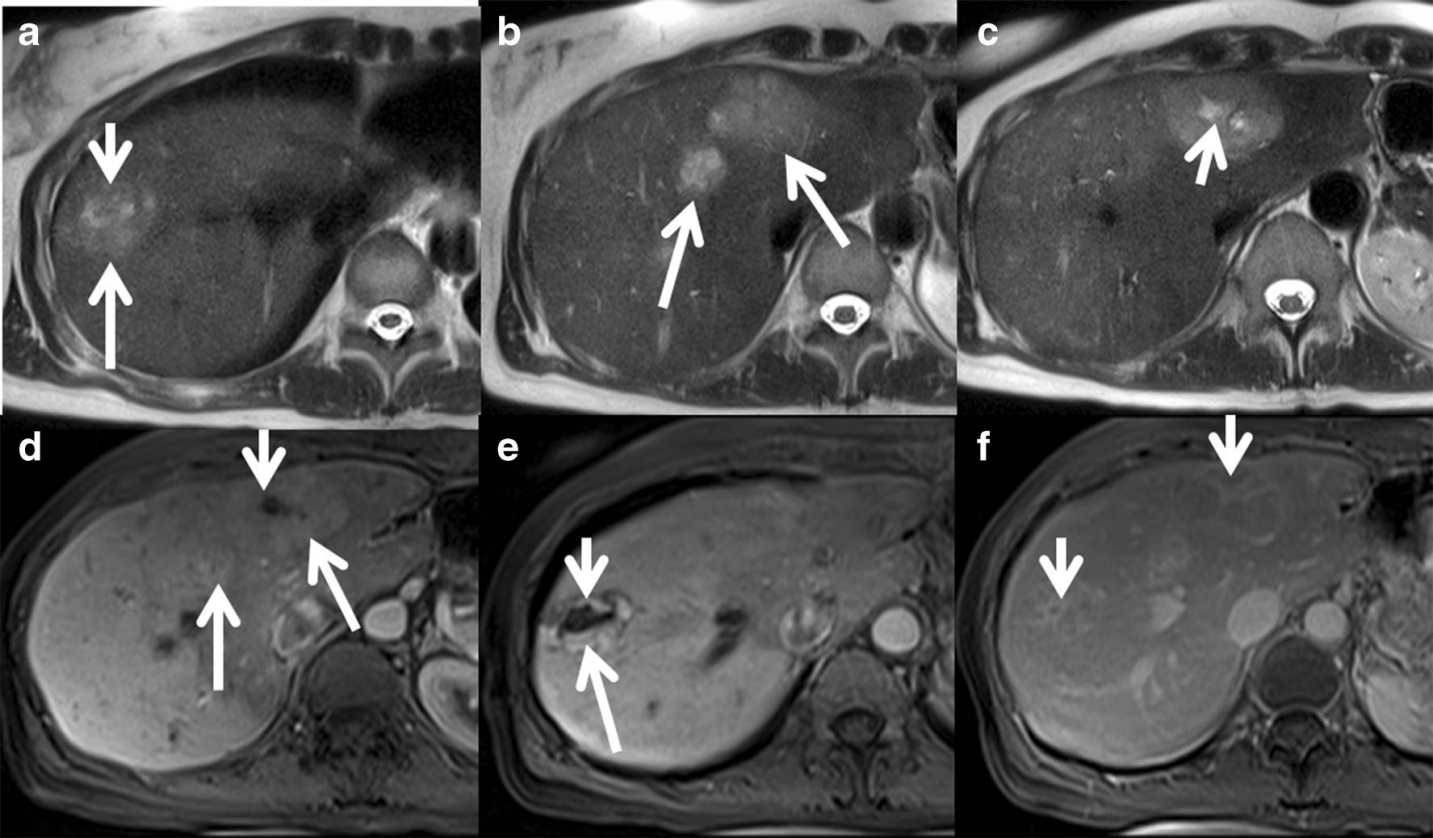
Hepatic adenomatosis in a 45-year-old male. (**a**), (**b**) and (**c**) Axial T2W MRI at multiple levels shows at least three mildly T2 hyperintense focal lesions (*long arrows*) in the right and left lobes of the liver with vague central T2 hyperintense areas (*short arrows*). (**d**) and (**e**) Axial post-contrast MRI in the arterial phase shows moderate enhancement of the lesions (*long arrows*) with central non-enhancing areas (*short arrows*). (**f**) Axial post-contrast MRI in the delayed phase shows no persistent enhancement with mild delayed enhancement of central areas (*short arrows*). Ultrasound-guided biopsy showed β-catenin-mutated hepatocellular adenoma with no dysplasia. The patient is planned for liver transplantation

Hepatic adenomatosis has been defined as the presence of multiple adenomas (arbitrarily >10) without any history of steroid therapy or glycogen storage disease [48]. It occurs in females during the 4th and 5th decades of life. Proposed aetiologies include congenital or acquired hepatic vascular abnormalities, mutations of the *HNF1A* gene and non-alcoholic fatty liver disease. HCAs in patients with hepatic adenomatosis may be of the inflammatory, HNF-1a-mutated or β-catenin-mutated subtypes, and their imaging appearances may vary accordingly [49] (Fig. 10). Contrary to the popular belief of increased complications in hepatic adenomatosis, a recent meta-analysis showed that hepatic adenomatosis per se does not have any increased risk of complications; the tumour size and subtype determine the risks of malignancy and bleeding [50]. Associated MODY should be evaluated in patients with hepatic adenomatosis and liver imaging is recommended in the relatives [51].

## Role of hepatobiliary-specific contrast

The appearance of FNH on hepatobiliary contrast has been discussed previously in the article. As FNH is a do-not-touch lesion and HCAs often warrant surgery, differentiating both of them, also from malignant hepatic lesions, is of prime importance. Hepatobiliary contrast is now being increasingly used in atypical cases for differentiation, thereby avoiding invasive biopsies in a good number of cases.

**Fig. 11:**
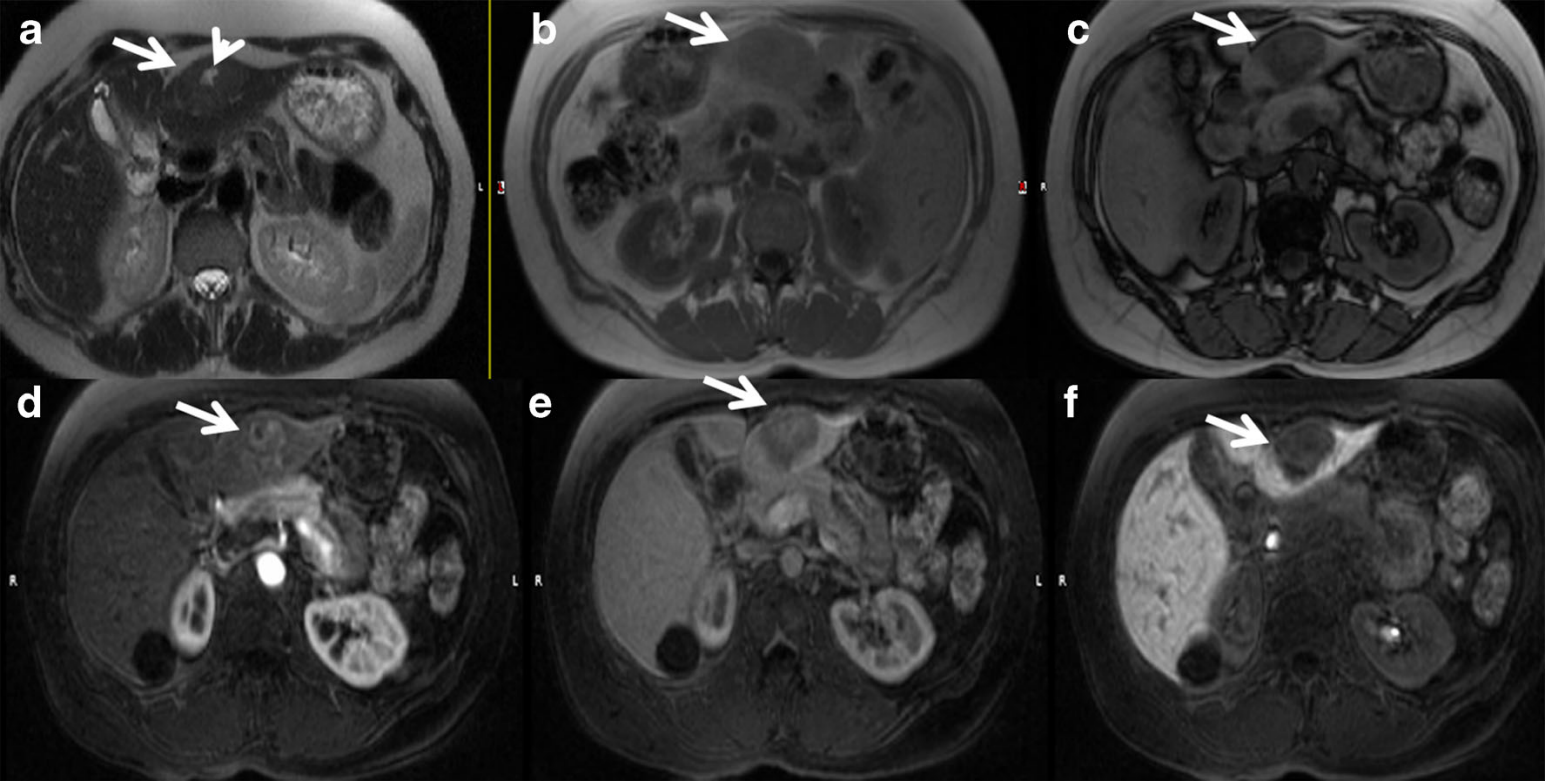
A 43-year-old female with typical hepatocyte nuclear factor 1alpha (HNF-1alpha)-inactivated hepatocellular adenoma. Axial T2W HASTE image (**a**) demonstrates a well-circumscribed mass in the lateral segment, which is only minimally hyperintense to the adjacent liver parenchyma (*white arrow*). There is a focal region of more increased T2W signal centrally (*arrowhead*). Axial in (**b**) and opposed (**c**) phase T1W dual echo GRE images demonstrate homogeneous loss of signal intensity within the lesion (*white arrows*) but no SI drop in the adjacent liver. Note misregistration between (**b**) and (**c**) as images were acquired in separate breath-holds. Axial fat-suppressed T1W GRE images after the injection of gadoxetic acid during the early arterial (**d**), portal venous (**e**) and 20-min hepatobiliary (**f**) phases demonstrate heterogeneous arterial enhancement with lack of persistent enhancement on portal venous phase and no uptake of gadoxetic acid during the hepatobiliary phase (*arrows*)

HCAs show mild-to-moderate arterial enhancement in contrast to intense enhancement in FNH. However the inflammatory subtype can show arterial enhancement similar to FNH. The majority of HCAs (93 %) become hypointense on portal venous and delayed hepatobiliary phases with gadoxetic acid, unlike FNH (Fig. 11). The absence of biliary ducts in adenomas, and therefore reduced biliary excretion mechanisms, is believed to be one of the factors responsible for the decreased uptake. Again the inflammatory subtype can show mild hyperintensity on hepatobiliary phase possibly due to pooling of the contrast agent within the peliotic areas [18, 52]. Perhaps the most important recent discovery regarding inflammatory HCA is that it can mimic FNH on MRI. Thomeer et al. and Agarwal et al. both identified that 25 and 46 % of inflammatory HCAs are iso-hyperintense on the hepatobiliary phase on gadoxetic acid-enhanced MRI [53, 54]. In addition, they often mimicked FNH on other imaging sequences. These two recent publications should caution radiologists when using hepatocyte-specific contrast agents to diagnose FNH; if patients are at high risk for the inflammatory type of HCA (obesity, alcohol use), it may not be appropriate to rely on imaging features alone to make a diagnosis of FNH.

## Complications

The two major complications of HCAs are *(a)* intratumoral bleeding with or without associated rupture and haemoperitoneum and *(b)* malignant transformation to HCC. Different subtypes of HCA show variable complication rates.

**Fig. 12:**
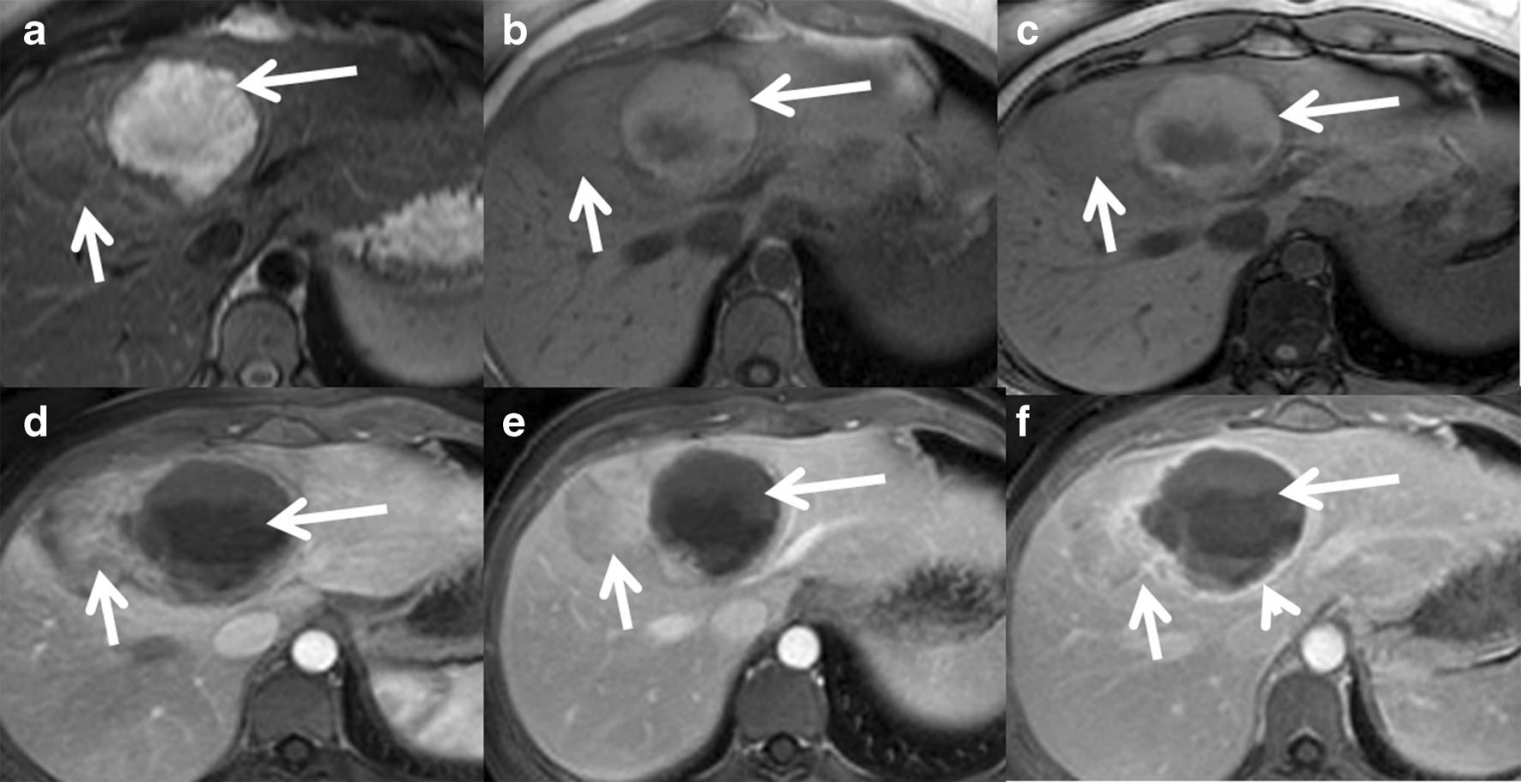
Hepatocellular adenoma with haemorrhage in a 40-year-old female on oral contraceptive pills. (**a**) Large heterogeneous lesion involving segment 8 and 4 of the liver showing a well-defined T2 hyperintense area along its medial aspect (*long arrow*) and T2 isointense area along its lateral aspect (*short arrow*). Axial T1 in-phase (**b**) and out-of-phase (**c**) MRI shows heterogeneous T1 hyperintensity in the medial portion (*long arrow*) without signal drop representing subacute haemorrhage. Axial post-contrast MRI shows heterogeneous mild-to-moderate enhancement of the lateral part of the lesion (*short arrow*) in the arterial phase (**d**) becoming isointense on venous (**e**) and delayed phases (**f**). The haemorrhagic component in the medial part of the lesion (*long arrow*) shows only peripheral capsular enhancement (*arrowhead*)

Haemorrhage can occur in 15–20 % of HCAs [55]. Bleeding can be intratumoral, intrahepatic or extrahepatic (Fig. 12). Risk factors for bleeding of HCA include a diameter of 35 mm or more, visualisation of central and peripheral intralesional arteries, location in the left lateral liver and exophytic growth [56]. Although peliosis is much more common in the inflammatory subtype compared to the HNF 1-alpha subtypes (52 % vs. 4 %), there is no notable difference in the risk of clinically manifest bleeding between these two subtypes (16 % vs. 9 %) [33]. Bleeding is very rare in the β-catenin subtype although the exact incidence is unknown.

Malignant transformation of hepatic adenoma to hepatocellular carcinoma has been variably reported to occur in 5–10 %, and a recent meta-analysis showed the risk of malignant transformation to be 4% in females and 47 % in males [57, 58]. The important risk factors for malignant transformation of HCAs are male sex, obesity, concomitant glycogen storage disease, anabolic steroid usage, β-catenin–mutated subtype and tumours larger than 5 cm in maximum dimension. β-Catenin-mutated HCAs show the highest risk of malignancy among all the subtypes. Two-thirds of HCAs with malignant transformation show β-catenin mutation and one-third display cell atypias. Also metabolic syndrome is an emerging condition and has been associated with malignant transformation of HCA in males [57]. Hepatocellular carcinomas may develop either as a macroscopic nodule larger than 1 cm in maximum dimension or as multiple microscopic foci. Approximately 10 % of the inflammatory subtype can show additional β-catenin activation and may progress to HCC [36].

## Management

Recent classification of HCAs into different subtypes has allowed better understanding of the natural history and biological behaviour of these relatively rare tumours. This has great implications for the imaging and management of these tumours. Many of the previously followed treatment strategies such as surgical resection for all hepatic adenomas and liver transplantation for multiple adenomas are now changing as we have better insight into these tumours because of recent advances in molecular genetics and imaging [35]. Early recommendations on management were made by Bioulac-Sage et al. based on the clinical features, gender, molecular subtype and imaging features [39].

**Fig. 13:**
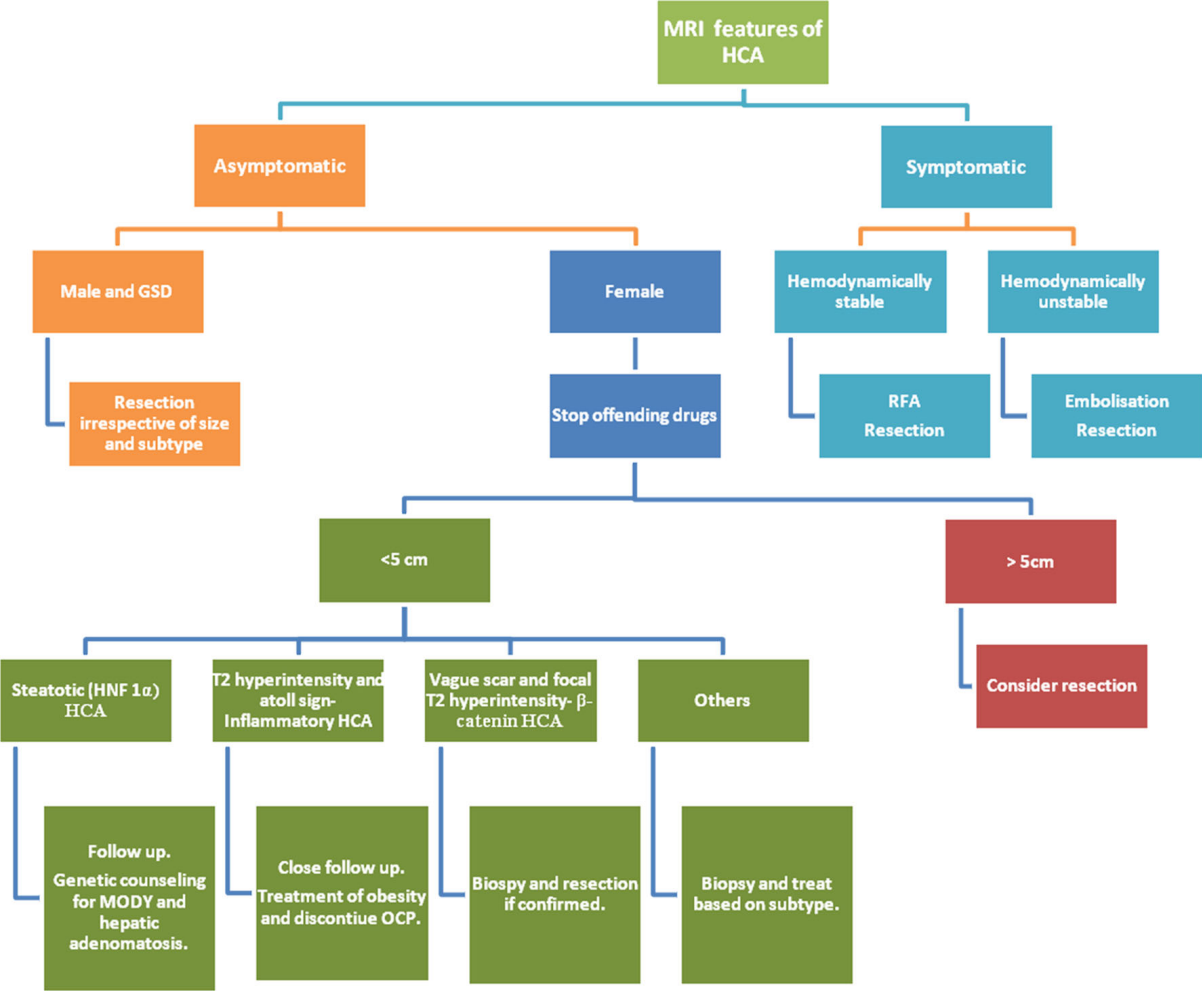
The algorithmic approach to the management of hepatocellular adenoma (HCA) based on clinical features, gender, imaging and histology

The management strategy is broadly based on the presence or absence of symptoms (Fig. 13). Incidental depiction of hepatic adenoma in an asymptomatic patient needs subtype classification based on MRI as steatotic (corresponding to HNF 1-alpha) and heterogeneous non-steatotic (inflammatory, β-catenin and unclassifiied) hepatic adenomas. Steatotic HCAs have no significant malignant risk; the risk of bleeding is low and hence can be managed by clinical and imaging follow-up without resection or need of biopsy. Heterogeneous non-steatotic HCAs larger than 5 cm, HCAs that continue to grow after stopping use of the offending drugs, HCAs with β-catenin activation, HCAs with malignant changes and all HCAs in males need surgical resection. Resection is effective, with low rates of recurrence [36, 39]. Biopsy is recommended for non-steatotic HCAs less than 5 cm to look for β-catenin mutation, which has a high risk of malignant transformation and needs resection.

In symptomatic HCAs treatment depends on the duration and type of symptoms. Patients who are haemodynamically unstable because of intra- or extrahepatic bleeding require immediate treatment with either hepatic artery embolisation or surgery [59]. Conservative treatment may be considered in haemodynamically stable patients followed by elective definite management, which can be surgery, chemoembolisation or RFA. Trans-arterial chemoembolisation reduces the risk of bleeding during elective surgery and may avoid surgical resection. Radiofrequency ablation is an effective, less invasive approach for (1) tumours smaller than 4 cm in maximum dimension, (2) patients who are not surgical candidates and (3) those who prefer to avoid surgery after discussion and full understanding of the available treatment options. Radiofrequency ablation has been found to be the most cost-effective approach in the management of small HCAs as compared with surgery, transarterial embolisation and watchful waiting [60].

There are no established guidelines on the optimal interval and duration of follow-up. Yearly imaging surveillance of HCAs is recommended for both solitary HCA and hepatic adenomatosis, and some authors recommend periodic surveillance until menopause [40].

Management of hepatic adenomatosis should depend on the underlying histological subtype and size of the lesions rather than on their number. Symptomatic lesions and asymptomatic lesions larger than 5 cm require surgical resection. Liver transplantation is recommended if there is progressive liver failure or malignant transformation and is no longer advised for patients with asymptomatic familial adenomatosis [61]. Genetic counselling for MODY3 is recommended. Management of HCAs in patients of child-bearing age is complicated because of the possible increased risk of bleeding and growth during pregnancy. Overall treatment strategies are similar to those in non-pregnant females; however close clinical and imaging monitoring is recommended for early detection of complications that may need aggressive management because of increased foeto-maternal morbidity [62].

## Conclusion

Recent advances in the molecular genetics and genotype-phenotype correlations in benign hepatocellular tumours have greatly improved our understanding leading to subtype recognition and better differentiation from other hypervascular lesions. Each of these tumour subtypes has distinct genetic mutations, molecular abnormalities, histopathological features, imaging findings, biological behaviour and prognosis. FNH typically does not bleed or undergo malignant change. HCAs with β-catenin mutations frequently undergo malignant change, inflammatory HCAs commonly bleed, and HNF-1α HCAs typically show a favourable prognosis. Many of these have reasonably specific imaging features permitting accurate diagnosis, which has significant implications for their management (Tables 1 and 2).

**Table 1:** Summary of clinical features, demographics, histopathology, immunohistochemistry and molecular genetics of major subtypes of hepatocellular adenoma (HCA) and focal nodular hyperplasia (FNH)

	Subtype	Frequency	Gender	Genetic mutation	Immunohistochemical marker	Histological phenotype	Clinical association	Complications
Focal nodular hyperplasia	Classic	80 %	F> > M	Angiopoietin 1 and 2. β-Catenin activation without mutation	Glutamine synthetase (perivenous)	Abnormal nodular architecture, malformed vessels and cholangiolar proliferation	Hereditary haemorrhagic telangiectasia, congenital absence of portal vein, haemangiomas	Very low risk of haemorrhage. No risk of malignant transformation
	Non-classic	20 %	F> > M	Angiopoietin 1 and 2. β-Catenin activation without mutation	Glutamine synthetase (perivenous)	Cholangiolar proliferation with either abnormal nodular architecture or malformed vessels	Multiple FNH syndrome associated with haemangiomas, cyst and adenoma	Slightly increased risk of bleeding in telangiectatic subtype. False risk of malignancy in inflammatory hepatic adenomas misclassified as ‘telangiectatic FNH’
Hepatocellular adenoma	Inflammatory HCA	40–55 %	F> > M	IL6ST (60 %), STAT3 (40 %)	Serum amyloid A (SAA) and C-reactive protein (CRP)	Intense polymorp infiltrates, marked sinusoidal dilatation and thick-walled arteries	Obesity. hepatic steatosis, high alcohol intake and inflammatory syndrome	Increased tendency to bleed. 10 % express β-catenin and are at risk of malignant transformation
	HNF1α-mutated HCA	30–35 %	Exclusively F	TCF1	Lack of LFABP1 expression	Intracellular lipid	Adenomatosis and MODY 3 diabetes	Lower tendency to bleed and very low risk of malignancy. Best prognosis.
	β-Catenin-mutated HCA	10–15 %	F > M	CTNNB1	Glutamine synthase (diffusely positive) and β-catenin	High nuclear-cytoplasmic ratio, nuclear atypia and acini formation	Male hormone and glycogen storage disease	Increased risk of malignant transformation
	Unclassified	10 %	–	–	–	–

**Table 2:** Summary of MRI features of major subtypes of hepatocellular adenoma (HCA) and focal nodular hyperplasia (FNH)

	Subtype	T1W	T2W	In and out of phase	Arterial	Portal venous/delayed	Remarks
Focal nodular hyperplasia	Classic	Iso- to mildly hypointense	Iso- to mildly hyperintense	No signal drop	Intense enhancement	Persistent enhancement	T2 hyperintense scar showing delayed enhancement
	Non-classic	Heterogeneous hypo- or iso- or hyperintense	Heterogeneous iso- or hyperintense	Focal signal drop rarely	Intense heterogeneous enhancement	Heterogeneous persistent enhancement with pseudocapsule	Absent or atypical scar
Hepatocellular adenoma	Inflammatory HCA	Iso- to mildly hyperintense	Diffusely hyperintense	No diffuse signal drop	Intense enhancement	Persistent enhancement	Atoll sign-T2 hyperintense rim. 10 % can have focal intratumoral fat
	HNF1a-mutated HCA	Iso- to hyperintense	Iso- to mildly hyperintense	Diffuse signal drop	Moderate enhancement	No persistent enhancement	
	ß-catenin-mutated HCA	Isointense	Iso-, hypo- or hyperintense	No diffuse signal drop	Intense	Variable. May show portal venous washout	Faint scar and ill-defined T2 hyperintense foci
	Unclassified	–	–	–	–	–	
